# Higher-Level Cognitive Function and Obstacle Attributes: An fNIRS Study in Older Adults With Parkinson’s Disease

**DOI:** 10.1093/geroni/igaa057.858

**Published:** 2020-12-16

**Authors:** Jeffrey Hausdorff, Topaz Sharon, Ilan Kurz, Hagar Bernad-Elazari, Ira Galperin, Nir Giladi, Anat Mirelman, Inbal Maidan

**Affiliations:** 1 Tel Aviv Sourasky Medical Center, Tel Aviv, Tel Aviv, Israel; 2 Tel Aviv University, Tel Aviv, Israel; 3 Ben Gurion University, Beer Sheva, Israel; 4 Tel Aviv Sourasky Medical Center, Tel Aviv, Israel

## Abstract

Older adults with Parkinson’s disease (PD) rely on prefrontal activation to compensate for impaired motor function during the performance of complex mobility-related activities such as obstacle negotiation. However, the influence of the properties of the obstacles on prefrontal activation has not been systematically evaluated. Here, we examined the effects of obstacle height and anticipation time on prefrontal activation in patients with PD and older adults. 34 patients with PD (age: 67.4±5.7 years; 14 women) and 26 older adult controls (age: 71.3±8.9 years; 11 women) walked in an obstacle course while negotiating anticipated and unanticipated obstacles at heights of 50 mm and 100 mm. Prefrontal activation was measured using functional Near-Infrared Spectroscopy (fNIRS); obstacle negotiation performance was measured using Kinect cameras. PD patients showed greater increases in prefrontal activation during and after obstacle crossing compared to the older adults (p<0.001). Obstacle height affected prefrontal activity only when crossing anticipated obstacles (time x height interaction, p=0.011); in that case, higher obstacles were accompanied by higher prefrontal activity. PD patients showed higher levels of activation during unanticipated obstacles, compared to anticipated obstacles (p=0.015). Different correlations between prefrontal activation and obstacle negotiation strategies were observed in the patients and the controls. These results point to the use of prefrontal activation as a compensatory mechanism in PD. Moreover, the higher activation of prefrontal regions during more challenging obstacles suggests that there is a greater reliance on cognitive resources in these demanding situations that may contribute to the higher risk of falls in patients with PD.

